# Evaluation of a community intervention program on knowledge, attitudes, and practices regarding severe fever with thrombocytopenia syndrome in Anhui Province, China

**DOI:** 10.3389/fpubh.2022.891700

**Published:** 2022-10-31

**Authors:** Lei Gong, Yong Zhang, Jinsheng Wang, Yingying Xiong, Jiling Wang, Jiabing Wu, Fang Chen, Meng Zhu, Donglin Cheng, Xuqin Jiang

**Affiliations:** ^1^Department of Acute Infectious Disease Prevention and Control, Anhui Provincial Center for Disease Control and Prevention, Hefei, China; ^2^Department of Acute Infectious Disease Prevention and Control, Chuzhou Municipal Center for Disease Control and Prevention, Chuzhou, China; ^3^Department of Acute Infectious Disease Prevention and Control, Anqing Municipal Center for Disease Control and Prevention, Anqing, China; ^4^Department of Pediatrics, Affiliated First Hospital, University of Science and Technology of China (USTC), Hefei, China; ^5^Department of Respiratory and Critical Medicine, The Second People's Hospital of Hefei, Hefei, China; ^6^Department of Respiratory and Critical Medicine, Affiliated First Hospital, USTC, Hefei, China

**Keywords:** intervention, knowledge, attitude, practice, severe fever with thrombocytopenia syndrome

## Abstract

**Background:**

Severe fever with thrombocytopenia syndrome (SFTS) is a novel infectious disease with no specific therapeutics and vaccines. We hypothesize that health education in vulnerable people would ameliorate their knowledge, attitudes and practices (KAP) regarding SFTS and reduce its prevalence.

**Methods:**

A four-stage cluster cross-section study in sixteen community units was performed. Sixteen groups were allocated to the intervention or control groups. A 6 months education program was administrated. The primary outcome was KAP scores 6 months after intervention. Predictors of KAP score changes were also analyzed.

**Results:**

Eight hundred and fifteen valid questionnaires pre-intervention and 767 ones post-intervention were retreated. No significant differences were found in demographic characteristics and KAP scores before intervention. A significant improvement in KAP score (16.8 ± 4.7 vs. 22.0 ± 4.2, *p* < 0.001) in the intervention group was observed compare with the controls. Educational level and intervention program were the common predictors of KAP score changes.

**Conclusions:**

Education improved KAP scores in SFTS vulnerable people which may contribute to the control of the disease.

## Introduction

Severe fever with thrombocytopenia syndrome (SFTS) is a novel infectious disease discovered in 2010 in China (1), and then in South Korea, and Japan (2, 3). In the following 6 years after the first report, STFS were reported in 23 provinces, distributed mainly in north-eastern, eastern, and central China (4, 5). Epidemiological investigation demonstrated that most patients were farmers or tea-pickers living in wooded and hilly regions (6). The majority of patients were farmers, who accounted for over 80.0% of the total infected population (7–9).

The pathogen of SFTS was proved to be a new kind of virus. The pathogen of SFTS was proved to be a new kind of virus, a member of the genus phlebovirus in Bunyaviridae family. The virus particle is spherical, 80–100 nm in diameter, with an outer lipid envelope and spinous processes on the surface (1). Researches demonstrated that the virus isolated from patient's blood was highly homological in nucleotide sequences to those found in *Haemaphysalis longicornis* (10, 11), and some of the patients had a clear exposure history of tick-bitten in the previous 14 days. These indicated that SFTS was an insect-mediated contagious disease and tick bite was the main contagious way of the disease. In addition, person-to-person transmission was also occasionally reported. Blood, other body fluid, vomit contents, and probably aerosol contacts were also confirmed as the ways of secondary transmissions (12–14).

The clinical manifestations varied individually in the patients. Some cases had mild symptoms only while in the others it could be severe or even fatal. At present, anti-viral drugs such as ribavirin, glucocorticoids, doxycycline, plasma replacement, transfusion of platelets and gamma globulin, etc are usually tried for SFTS treatment. However, with these measures, the disease could remain to be severe or even fatal in some patients. Apparently, other ways of controlling and preventing should be exploited.

In controlling epidemic disease, education has been proved useful as an alternative method. For example, study assessing the knowledge, attitudes and practices (KAP) levels on zoonotic disease proved education effective (15). As SFTS is mainly transmitted through tick bites, effective personal protection knowledge, proper attitude and behavior can theoretically reduce the chance of tick bites to some extent, thus reducing the probability of SFTSV infection. Considering the fact that most of SFTS patients were farmers living in the hilly areas, we conducted a SFTS-related education program to see if SFTS knowledge and personal protective ability of the vulnerable people were improved. Here we report the effectiveness of a half-year intervention program about SFTS. We hope these findings be useful for developing public health strategies for SFTS prevention and controls.

## Materials and methods

### Setting and participants

**Figure 1:**
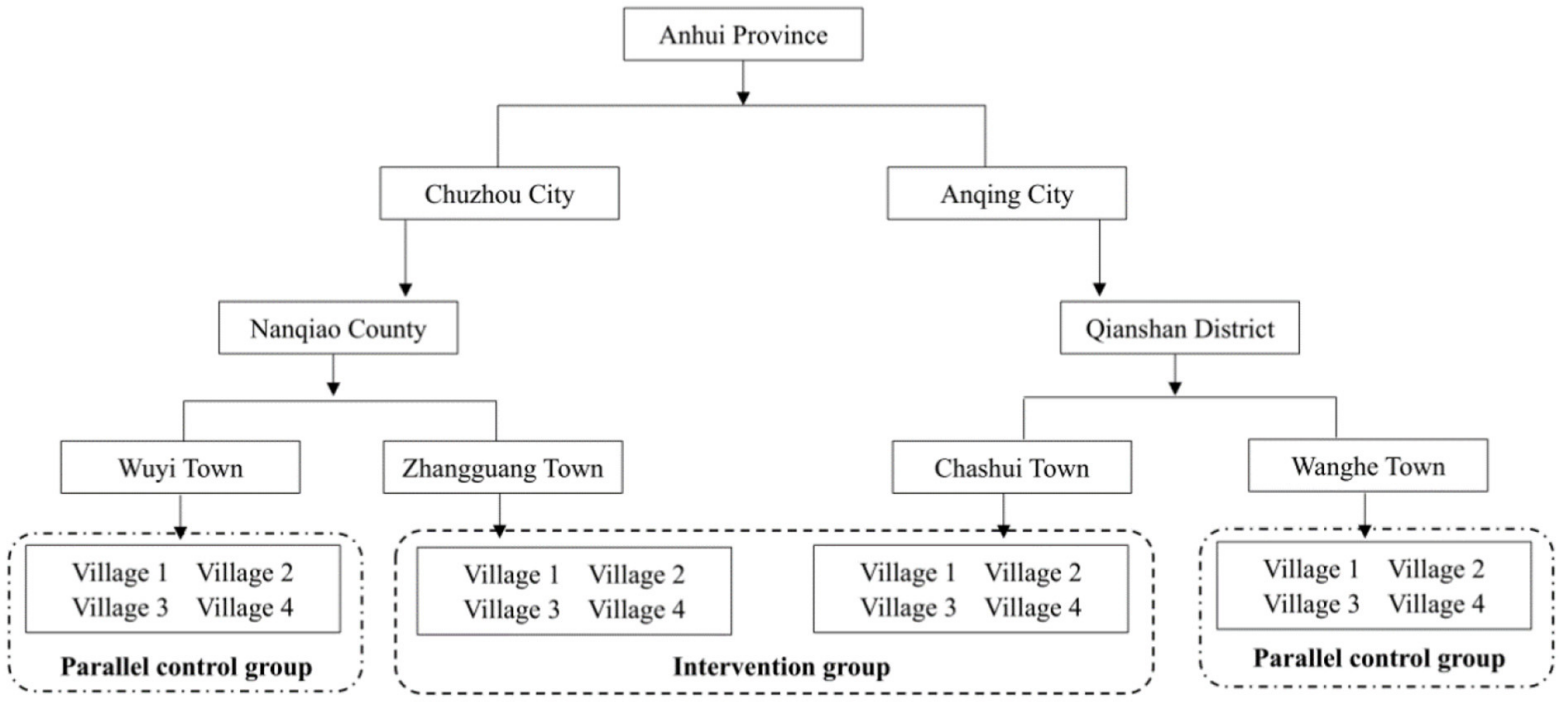
Intervention diagram of this study.

SFTS remains a hazard epidemic disease to the residents in Anhui Province. From 2010 to 2016, a total of 1250 cases of SFTS were reported, including 328 cases in Anqing City and 200 cases in Chuzhou City. Farmers were the dominated in occupational distribution, with 1046 reported cases, accounted for 83.7%. In this study, a four-stage cluster sampling technique was used to decide the intervention setting and participant recruitment. First, the average annual SFTS incidence from the year 2010 to 2016 were sequenced at the municipal level of Anhui Province, and the first (Anqing city) and the second (Chuzhou city) sites were chosen. Then, the county and district were sorted in Anqing city and Chuzhou city, and the first (Qianshan county and Nanqiao district) were chosen. In the same way, Chashui town and Zhangguang town were chosen, and two towns, Wanghe town of Qianshan county and Wuyi town of Nanqiao district were selected as parallel control town by economic, landform, demographic characteristics, and labor habit. Four villages of each intervention town were selected by the accumulated reported SFTS case sequences, while four villages of each parallel control town were also recruited. Consequently, 16 villages were chosen. Of them, eight villages from Zhangguang town and Chashui town were set as the intervention groups, while eight other villages of Wuyi town and Wanghe town were set as the parallel control groups (Figure 1).

### Intervention measures

This structured education program for vulnerable population in endemic areas was conducted from June, 2017 to December, 2017. The education program was carried out in the selected villages, while no intervention measures were conducted in the control group villages. The program was tailor-made according to the specific needs of the rural residents and school students, and performed in community and individual levels. The intervention measures included (1) SFTS knowledge spreading to the community population by posters, banners and propaganda brochures, which were designed according to expert consultations; (2) Primary school students training. In which, students were trained by health education lectures and peer discussions and encouraged to spread SFTS knowledge to their families; (3) Individual counseling and interaction provided by the local clinicians for persons who exhibited lower awareness of SFTS in the pre-intervention phrase; (4) SFTS knowledge spreading by mass media including radio, television, newspapers and internet.

## Evaluation of the intervention program

### Study size

The calculation of the sample size and sampling diagram for present study adopted the following equation.


$$\begin{array}{l} n=\frac{{\left(u_{\alpha }+u_{\beta }\right)}^{2}2p\left(1-p\right)}{{\left(p_{1}-p_{2}\right)}^{2}}\\ \;=\frac{{\left(2.58\;+\;2.58\right)}^{2}\times 2\;\times \;0.45\times \left(1-0.45\right)}{{\left(0.35-\;0.55\right)}^{2}}=329 \end{array}$$

where *n* = the sample size; u_α_ = 2.58 (the standard normal deviate when the α was set as 0.01); u_β_ = 2.58 (the standard normal deviate when the 1–β was set as 0.01); *p*_1_ = 0.35 (the knowledge rate on SFTS of control group was calculated as 35.0% by a pilot survey); *p*_2_ = 0.55 (the expected knowledge rate on SFTS after intervention program); $p=\frac{\left(\;p_{1}+p_{2}\right)}{2}=\frac{\left(\;0.35\;+\;0.55\right)}{2}$ = 0.45.

The sample size was increased by 10% to compensate the information loss because of incorrect, incomplete and inevitable missing questionnaires during data collection. Consequently, the required sample size of each pre-intervention group, post-intervention group, pre-control group, and post-control group was at least 362 participants.

### Participants and data collection

Participant eligibility criteria for participants in this study were 18 years old and over, living in the district more than 6 months; primary school students of grade 4–6 or junior high school students in the chosen towns. Data were collected at pre-intervention and the end of 6 months.

### Questionnaire design

A standardized questionnaire was designed according to “The diagnosis and treatment programs of severe fever with thrombocytopenia syndrome” (16). It contains four parts: (1) demographic characteristics including age, gender, occupation, educational level, et al.; (2) six questions evaluating the SFTS knowledge levels; (3) four and six questions respectively; (4) the attitudes and practice on SFTS. The validity of this questionnaire was confirmed by a Cronbach's alpha (α) internal consistency coefficient of 0.79 from the second to the fourth parts by the pilot survey in a village of Anqing city involving 66 participants.

### Scoring

Scoring assignment and calculation referred to the methods of literatures, and the responses to the questionnaire questions with right and wrong were scored as 1 and 0, respectively. When the answers were with the trend of correctness, the responses were scored as 0, 1 and 2 (17). The calculated maximum score of knowledge, attitude, practice and total were 14, 8, 8, and 30 for one participant, respectively. The P_90_ value of knowledge score, attitude score and practice score were calculated and set as the standard to categorize for good and poor KAP score, the KAP score equal and >P_90_ were defined as good, otherwise, defined as poor.

The knowledge rate was calculated as following equation:


$$\begin{array}{l} Y\left(\%\right)=\frac{M}{N}\times 100=\frac{\sum m}{14n}\times 100 \end{array}$$

Where Y = the knowledge rate; M = the total number of correct responses = ∑m = Sum(m_1_, m_2_….m_n_); m = the number of correct responses of knowledge questions of one participant; N = the total number of questions; 14 = the full marks of knowledge questions; *n* = the number of participants.

### Statistical analysis

EpiData software, version 3.1 (a freeware distributed by EpiData Association, Odense, Denmark, available for download at: http://www.epidata.dk/download.php) was used to enter the data twice independently. Statistical analysis was performed with SPSS software, version 11 (SPSS, Chicago, IL, USA). Participants' demographic characteristics and the some responses in intervention and control groups were described using frequency and proportions for qualitative characteristics, and the qualitative values were compared using a chi-square test. The knowledge, attitude and practice scores were described using means ± standard deviations (SDs), and a two-sample *t* test was used for the continuous variables. Association between variables related to participants' characteristics and intervention effects was tested by simple and multiple regression models. Only variables meeting the statistical *p* value threshold of < 0.1 in univariate analysis were included in the multivariable model, the forward step-wise method of multiple regression analysis was used to further explore the influence factors of intervention. Level of significance (α) was set at 0.05.

### Ethical considerations

Ethical approval was obtained from Anhui Provincial Center for Disease Control and Prevention, and informed consent was received from each of the participants or their guardian before enrolling in this study.

## Results

### Demographic characteristics of the participants

A total number of 768 valid questionnaires including 377 questionnaires from the intervention group and 391 questionnaires from the control group were collected in May, 2017. After the 6 months intervention, 386 valid questionnaires from the intervention group and 386 from the controls in the same 8 villages were collected (Figure 1). Results showed no significant differences between the invention and control groups in variables including region (*x*^2^ = 3.163, *p* = 0.075), gender (*x*^2^ = 2.442, *p* = 0.118), age (*x*^2^ = 0.045, *p* = 0.832), occupation (*x*^2^ = 0.045, *p* = 0.832), education level (*x*^2^ = 1.202, *p* = 0878) and frequency of outdoor work during the last months (*x*^2^ = 0.858, *p* = 0.835). See Table 1.

**Table 1:** Demographic characteristics of the intervention and control population.

**Variables**	**Intervention group**, ***n*** **(%)**		***P* value**	**Control group**, ***n*** **(%)**		***P* value**
	**Pre-test**	**Post-test**		**Pre-test**	**Post-test**	
	**(*n* = 377)**	**(*n* = 386)**		**(*n* = 391)**	**(*n* = 386)**	
**Region**			0.323			0.633
Qianshan county	201 (53.3)	192 (49.7)		187 (47.8)	178 (46.1)	
Nanqiao district	176 (46.7)	194 (50.3)		204 (52.2)	208 (53.9)	
**Gender**			0.368			0.759
Male	192 (50.9)	184 (47.7)		175 (44.8)	177 (45.9)	
Female	185 (49.1)	202 (52.3)		216 (55.2)	209 (54.1)	
**Age (years)**			0.631			0.331
Less than 18 years	180 (47.7)	191 (49.5)		199 (50.9)	183 (47.4)	
19 years and above	197 (52.3)	195 (50.5)		192 (49.1)	203 (52.6)	
**Occupation**			0.532			0.331
Student	179 (47.5)	192 (49.7)		199 (50.9)	183 (47.4)	
Community member	198 (52.5)	194 (50.3)		192 (49.1)	203 (52.6)	
**Educational level**			0.113			0.073
College/University	9 (2.4)	7 (1.8)		14 (3.6)	6 (1.6)	
10–12th grade	28 (7.4)	16 (4.1)		31 (7.9)	22 (5.7)	
7–9th grade	189 (50.1)	186 (48.2)		194 (49.6)	179 (46.4)	
1–6th grade	96 (25.5)	99 (25.6)		96 (24.6)	103 (26.7)	
No formal education	55 (14.6)	78 (20.2)		56 (14.3)	76 (19.7)	
**Frequency of outdoor work during the last month**			0.378			0.083
Usually (≧20 days)	39 (10.3)	48 (12.4)		42 (10.7)	53 (13.7)	
Often (10–19 days)	53 (14.1)	56 (14.5)		56 (12.8)	47 (12.2)	
Occasionally (1–9days)	150 (39.8)	131 (33.9)		178 (40.6)	124 (32.1)	
Never	135 (35.8)	151 (39.1)		162 (37.0)	162 (42.0)	

### Responses on SFTS knowledge

Correctness response rate of education participants was 12.0–50.7% before-intervention and 34.2–75.4% post-intervention. Significant changes in symptoms, route of transmission, and other knowledge were observed. Meantime, the correctness rate in knowledge ranged from 7.7–17.6% before intervention, and 6.7–21.5% after intervention. Comparatively, no significant differences were observed in the control groups. The participants with active attitude toward SFTS before intervention ranged from 43.0–77.5%, and participants were more active in attitude in dealing with the tick-bitten after intervention. Participants with appropriate behaviors ranged from 43.2–72.4%, and the correctness rate increased from 56.2–88.6%. Significant differences in practices before and after intervention were also observed (*p* < 0.001). The detailed data are shown in Table 2.

**Table 2:** Responses on SFTS of the study population.

**Variables**	**Intervention group**, ***n*** **(%)**		***P* value**	**Control group**, ***n*** **(%)**		***P* value**
	**Pre-test**	**Post-test**		**Pre-test**	**Post-test**	
	**(*n* = 377)**	**(*n* = 386)**		**(*n* = 391)**	**(*n* = 386)**	
**Knowledge**						
**Symptoms of SFTS**						
Fever	191 (50.7)	291 (75.4)	*P* < 0.001	69 (17.6)	83 (21.5)	0.176
Weakness, headache and muscle soreness	106 (28.1)	216 (56.0)	*p* < 0.001	64 (16.4)	81 (21.0)	0.099
Nausea, vomiting, anorexia and diarrhea	100 (26.5)	216 (56.0)	*p* < 0.001	45 (11.5)	62 (16.1)	0.066
Leucopenia	49 (13.0)	132 (34.2)	*p* < 0.001	34 (8.7)	26 (6.7)	0.306
Thrombocytopenia	68 (18.0)	174 (45.1)	*p* < 0.001	46 (11.8)	47 (12.2)	0.860
**Route of transmission**						
Contact with the blood and/or blood fluid directly	46 (12.2)	208 (53.9)	*p* < 0.001	64 (16.4)	57 (14.8)	0.538
Tick-bitten	57 (15.1)	176 (45.6)	*p* < 0.001	67 (17.1)	63 (16.3)	0.761
SFTS patients can be cured	94 (24.9)	156 (40.4)	*p* < 0.001	61 (15.6)	58 (15.0)	0.824
None vaccine against the SFTSV	58 (15.4)	197 (51.0)	*p* < 0.001	30 (7.7)	35 (9.1)	0.483
**Attitude**						
Use alcohol or iodine if bitten by tick	202 (53.6)	328 (85.0)	*p* < 0.001	168 (43.0)	167 (43.3)	0.933
Visit a doctor immediately if bitten by tick with symptoms	290 (76.9)	359 (93.0)	*p* < 0.001	303 (77.5)	259 (67.1)	0.001
Willing to consult	282 (74.8)	306 (79.3)	0.142	258 (66.0)	266 (68.9)	0.384
Willing to participate in free detection	234 (62.1)	261 (67.6)	0.109	232 (59.3)	213 (55.2)	0.242
**Practice**						
**Protective measures**						
Wore long-sleeved clothes	244 (64.7)	342 (88.6)	*p* < 0.001	233 (59.6)	223 (57.8)	0.607
Daub repellents	163 (43.2)	217 (56.2)	*p* < 0.001	137 (35.0)	125 (32.4)	0.434
Take protective measures when contact with the livestock	204 (54.1)	299 (77.5)	*p* < 0.001	200 (51.2)	193 (50.0)	0.748
Get rid of the ticks when finding in the livestock	273 (72.4)	333 (86.3)	*p* < 0.001	263 (67.3)	264 (68.4)	0.736
Sweep weed around house termly	259 (68.7)	317 (82.1)	*p* < 0.001	268 (68.5)	275 (71.2)	0.412

### Knowledge rate and KAP scores

Table 3 described the knowledge rate and KAP scores of the study population. The knowledge rate changed remarkably in the intervention group (39.1 pre- vs. 60.2% post-intervention respectively) while no significant change observed in the control group (pre- 30.6% vs. post-intervention 31.7%, respectively, *P* > 0.05). Similar changes were observed in pre- and post-intervention in knowledge score, attitude score.

**Table 3:** Differences in knowledge, attitude, practice, and overall score in between intervention and control groups.

**Variables**	**Intervention group**		***P* value**	**Control group**		***P* value**
	**Pre-test**	**Post-test**		**Pre-test**	**Post-test**	
	**(*n* = 377)**	**(*n* = 386)**		**(*n* = 391)**	**(*n* = 386)**	
Knowledge rate, %	39.1	60.2	*p* < 0.001	30.6	31.7	0.240
Knowledge score, $\bar{x}\pm s$	5.5 ± 2.2	8.4 ± 2.6	*p* < 0.001	4.3 ± 1.9	4.4 ± 2.0	0.295
Attitude score, $\bar{x}\pm s$	6.0 ± 1.8	7.0 ± 1.3	*p* < 0.001	5.5 ± 1.9	5.3 ± 2.3	0.154
Practice score, $\bar{x}\pm s$	5.3 ± 2.2	6.6 ± 1.6	*p* < 0.001	5.2 ± 2.2	5.2 ± 2.1	0.802
Total score, $\bar{x}\pm s$	16.8 ± 4.7	22.0 ± 4.2	*p* < 0.001	15.0 ± 4.5	15.0 ± 5.0	0.921

### Predictors of good KAP scores

Multivariate regression analysis showed that predictors of good knowledge were region, gender, education level, and intervention. The education level, frequency of outdoor work during the last month, and intervention education were the predictors of active attitudes. Age, education level, and intervention education were the predictors of appropriate practices. Education level and intervention were common predictors of KAP scores, see Table 4.

**Table 4:** Predictors of good knowledge, attitude, and practice score.

**Variable**	**Good, *n* (%)**	**Poor, *n* (%)**	**Crude OR (95%CI)**	**Adjusted OR (95%CI)**
**Knowledge**				
**Region**				
Qianshan county	62 (7.9)	720 (92.1)	1.0	1.0
Nanqiao district	116 (15.3)	642 (84.7)	2.098 (1.514–2.908)	2.258 (1.555–3.277)
**Gender**				
Female	75 (9.2)	737 (90.8)	1.0	1.0
Male	103 (14.1)	625 (85.9)	1.619 (1.181–2.221)	1.561 (1.078–2.262)
**Educational level**				
No formal education	19 (7.2)	246 (92.8)	1.0	1.0
1–6^th^ grade	37 (9.4)	357 (90.6)	1.342 (0.754–2.388)	1.443 (0.768–2.711)
7–9^th^ grade	102 (13.6)	646 (86.4)	2.044 (1.226–3.409)	2.031 (1.146–3.598)
10–12^th^ grade	11 (11.3)	86 (88.7)	1.656 (0.758–3.62)	2.713 (1.114–6.608)
College/University	9 (25.0)	27 (75.0)	4.316 (1.777–10.479)	6.34 (2.168–18.54)
**Intervention**				
No	43 (3.7)	1,111 (96.3)	1.0	1.0
Yes	135 (35.0)	251 (65.0)	13.897 (9.603–20.109)	16.09 (10.925–23.697)
**Attitudes**			
**Education level**				
No formal education	57 (21.5)	208 (78.5)	1.0	1.0
1–6^th^ grade	93 (23.6)	301 (76.4)	1.127 (0.776–1.639)	1.323 (0.888–1.97)
7–9^th^ grade	223 (29.8)	525 (70.2)	1.55 (1.112–2.161)	1.951 (1.358–2.803)
10–12^th^ grade	43 (44.3)	54 (55.7)	2.906 (1.769–4.773)	4.276 (2.517–7.265)
College/University	25 (69.4)	11 (30.6)	8.293 (3.85–17.864)	12.575 (5.655–27.961)
**Frequency of outdoor work during the last month**				
Never	154 (26.2)	434 (73.8)	1.0	1.0
Occasionally (1–9days)	156 (28.0)	402 (72.0)	1.094 (0.843–1.419)	1.047 (0.792–1.384)
Often (10–19 days)	74 (34.9)	138 (65.1)	1.511 (1.079–2.117)	1.613 (1.124–2.316)
Usually (≧20 days)	57 (31.3)	125 (68.7)	1.285 (0.894–1.848)	1.482 (0.998–2.2)
**Intervention program**				
No	247 (21.4)	907 (78.6)	1.0	1.0
Yes	194 (50.3)	192 (49.7)	3.71 (2.907–4.736)	4.216 (3.27–5.437)
**Practices**				
**Age (years)**				
Less than 18 years	229 (30.4)	524 (69.6)	1.0	1.0
19 years and above	105 (13.3)	682 (86.7)	0.352 (0.272–0.456)	0.323 (0.225–0.463)
**Education level**				
No formal education	18 (6.8)	247 (93.2)	1.0	1.0
1–6^th^ grade	56 (14.2)	338 (85.8)	2.274 (1.304–3.964)	1.263 (0.677–2.356)
7–9^th^ grade	219 (29.3)	529 (70.7)	5.681 (3.433–9.401)	2.927 (1.616–5.304)
10–12^th^ grade	27 (27.8)	70 (72.2)	5.293 (2.755–10.167)	7.377 (3.746–14.528)
College/University	14 (38.9)	22 (61.1)	8.732 (3.833–19.894)	12.072 (5.124–28.44)
**Intervention program**				
No	183 (15.9)	971 (84.1)	1.0	1.0
Yes	151 (39.1)	235 (60.9)	3.409 (2.633–4.414)	4.108 (3.099–5.445)

## Discussion

SFTS is a novel epidemic disease and no specific anti-SFTS virus drug and vaccine are available at present. Related health education might increase the knowledge of the disease in the vulnerable people and consequently reduce the morbidity. In this study, we performed a SFTS related education program and the results showed significant increases in KAP scores regarding of SFTS in the people experienced the education.

Educational intervention can improve the low knowledge, attitude and practice of a certain disease (18). Theoretically, the ameliorated knowledge about SFTS could modify personal daily behavior and reduce corresponding contagious risks. Adequate education could change the knowledge, attitude and practice of the vulnerable people. In fact, in the guideline and recommendations for preventing SFTS virus infection on vulnerable population in endemic areas, health education was one of the four recommended measures (16).

A former cross-sectional study reported that the knowledge rate of SFTS of community members and students in endemic areas was 56.2% (17). In the study, the knowledge rate was calculated on a question as “Do you know what SFTS is?”. Different from that study, the knowledge rate in our study was 30.6% before intervention. This difference was mainly due to the changes of investigation methods. In our study, 14 classified questions of knowledge were included and calculated which made the results more accurate and close to the reality.

The knowledge rate was 60.2% (increased by 21.1%) after intervention and knowledge score changed from 5.5 ± 2.2 to 8.4 ± 2.6 (*p* < 0.001) before and after intervention. Compared with the intervention group, the knowledge rate elevated only 1.1% in the controls. Similar changes were seen in the attitude score and practice score, as shown in Table 3. These results indicated that intervention could increase the knowledge about SFTS, change the subjects' attitude and practice significantly. These changes could potentially reduce the morbidity of SFTS.

In this study, we further explored the predictor of good KAP scores and the results showed that predictors of good knowledge rate were region, gender, educational level and intervention program et al., see Table 4. Among them, education and intervention were both common predictors to attitude and practice scores. Education was an independent factor affecting the knowledge score in our study, similar to the results of the other studies (15, 17, 18). It is common that people with higher education levels are more knowledgeable. The intervention program itself was also a common independent factor in this study. It remarkably increased the knowledge rate on SFTS. These results suggested that the intervention program could change participants' knowledge, attitude and practice, and thereafter the behavior, as proved in other studies (19–21).

This study provided a solid real world evidence of the affects of intervention on KAP regards SFTS. However, there were some limitations. First, the sample attendees were chosen with a limited group of villages. Second, an accurate assessment of the intervention itself was lacking. Which education method was most efficient was not assessed either. Third, we could not perform a follow up to assess the effects of intervention on the morbidity of SFTS directly duo to some limited conditions

In conclusion, education program could improve the knowledge, attitude and practice to SFTS in vulnerable people. Some factors such as personal education levels were predictors of good knowledge, active attitude and appropriate practices of SFTS. Education program as an intervention measure might be helpful in reducing the morbidity of SFTS.

## Data availability statement

The original contributions presented in the study are included in the article/supplementary material, further inquiries can be directed to the corresponding author.

## Ethics statement

Ethical approval was obtained from Anhui Provincial Center for Disease Control and Prevention, and informed consent was received from each of the participants or their guardian before enrolling in this study. Written informed consent to participate in this study was provided by the participants' legal guardian/next of kin.

## Author contributions

LG has obtained the approval of all other co-authors to submit this article. All authors have contributed to the manuscript.

## Funding

This study was supported by a grant from Anhui Provincial Health and Family Planning Commission (2017jk004), a grant from Anhui Provincial Science and Technology Department (1503062008), and a grant from National Health and Family Planning Commission of the People's Republic of China (201202006).

## Conflict of interest

The authors declare that the research was conducted in the absence of any commercial or financial relationships that could be construed as a potential conflict of interest.

## Publisher's note

All claims expressed in this article are solely those of the authors and do not necessarily represent those of their affiliated organizations, or those of the publisher, the editors and the reviewers. Any product that may be evaluated in this article, or claim that may be made by its manufacturer, is not guaranteed or endorsed by the publisher.
